# Beyond Kingpins: A Structural Approach to Cartel Violence Reduction in Mexico

**DOI:** 10.34172/ijhpm.9925

**Published:** 2026-08-09

**Authors:** Mohammad Karamouzian, Raul Bejarano-Romero, Felipe Krause, Jaime Arredondo Sanchez Lira, Dan Werb

**Affiliations:** ^1^Centre on Drug Policy Evaluation, MAP Centre for Urban Health Solutions, St. Michael’s Hospital, Toronto, ON, Canada.; ^2^Dalla Lana School of Public Health, University of Toronto, Toronto, ON, Canada.; ^3^HIV/STI Surveillance Research Center, and WHO Collaborating Center for HIV Surveillance, Institute for Futures Studies in Health, Kerman University of Medical Sciences, Kerman, Iran.; ^4^San Diego State University Research Foundation, San Diego, CA, USA.; ^5^Latin American Centre, Oxford School of Global and Area Studies, University of Oxford, Oxford, UK.; ^6^Canadian Institute for Substance Use Research, University of Victoria, Victoria, BC, Canada.; ^7^Division of Infectious Diseases and Global Public Health, University of California San Diego, La Jolla, CA, USA.

## A Tactical Success, Yet a Strategic Failure

 On February 22, 2026, Mexican security forces killed Nemesio Oseguera Cervantes (“El Mencho”), leader of the *Cártel Jalisco Nueva Generación*(CJNG; Jalisco New Generation Cartel), in a military operation in Tapalpa, Jalisco. Within hours, coordinated retaliatory violence erupted across more than 20 of Mexico’s 32 states: over 252 roadblocks, dozens of vehicles torched, at least 62 people killed, and major cities including Guadalajara and Puerto Vallarta paralyzed.^1^ International attention reflected the strategic significance of CJNG, the scale of the coordinated retaliation, and the disruption to civilian life and mobility. For millions of Mexicans, however, such violence is one of the defining features of everyday insecurity.

 The operation may be viewed as a tactical success given the removal of one of Mexico’s most powerful criminal leaders. Its aftermath, however, exposed a deeper strategic failure. Leadership decapitation can demonstrate the state’s coercive reach, satisfy domestic and bilateral demands for action, and temporarily disrupt a command structure.^2^ It does not, by itself, alter the economic, institutional, and transborder systems that allow criminal organizations to adapt, fragment, and reproduce coercive power.

 We argue that the central failure of the “kingpin strategy”—highly visible, politically expedient operations to target cartel leaders and dismantle criminal organizations^3^—is structural. It targets leadership while leaving intact the political economy that sustains cartel power,^4^ including diversified illicit and semi-licit revenues, corruption and state capture, coercive local governance, forced recruitment, weak civilian and judicial institutions, and cross-border demand and firearms flows (Figure).

**Figure:**
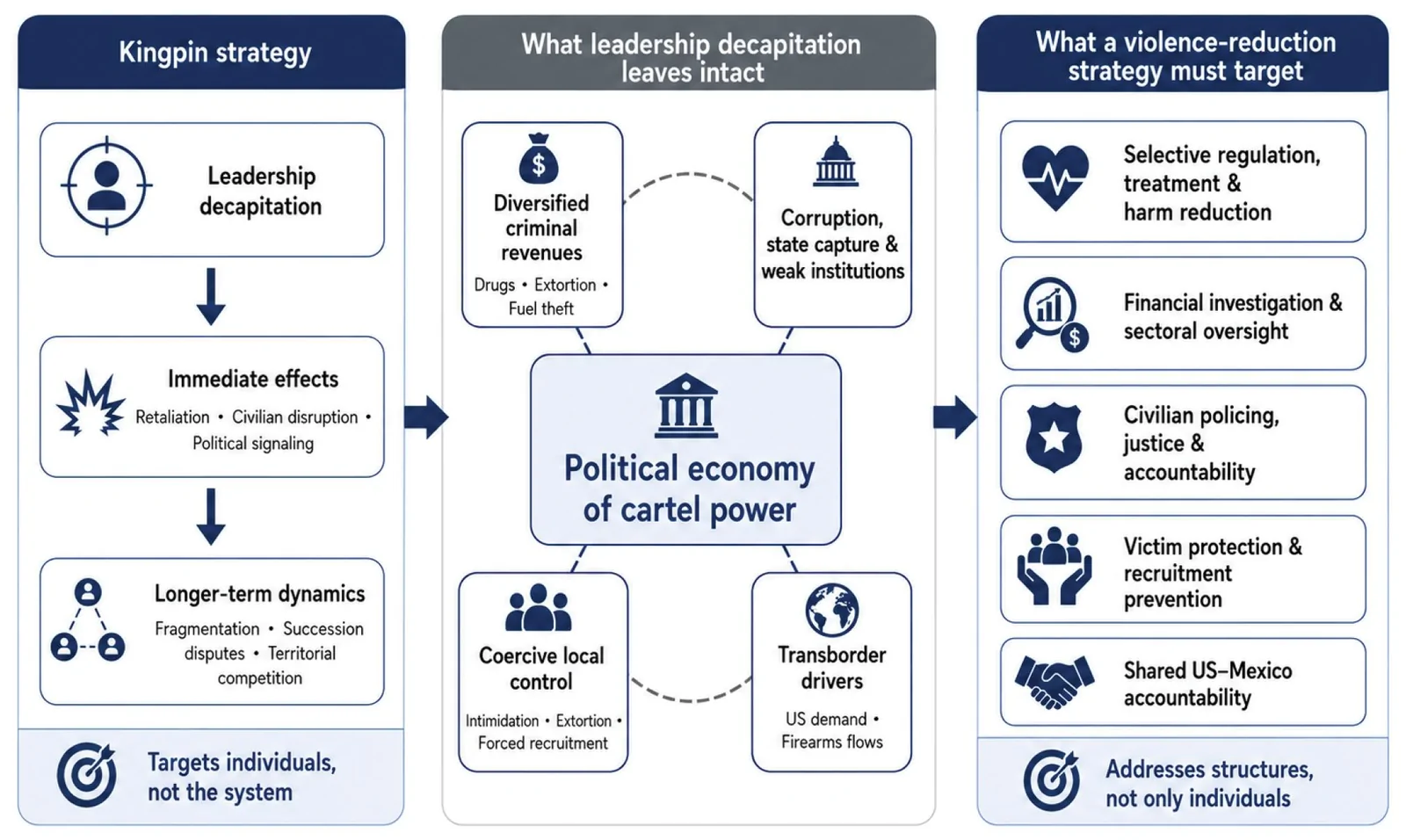
From Kingpin Decapitation to a Political Economy Approach to Cartel Violence Reduction.

## Why Leadership Decapitation Does Not Reduce Violence

 The kingpin strategy has shaped drug law enforcement across the Americas since the 1990s.^3^ Its logic is intuitive: remove the head, and the body collapses. Evidence from Mexico tells a different story. Kingpin removals have been associated with increases in homicides in affected municipalities, violent spillovers into surrounding regions, and disappearances, which have overwhelmed forensic institutions and created widespread family and intergenerational trauma.^5^

 These harms occur on different timelines. Some are immediate, including retaliation, roadblocks, arson, attacks on security forces, and acute civilian disruption, while others unfold over months and years, as surviving cells, rivals, and local allies recalibrate territorial control, succession arrangements, and market access. Leadership decapitation can trigger organizational fragmentation, producing smaller and more unpredictable groups that compete violently for territory and trafficking routes, protection rackets, and extortion markets.^2,6^ Indeed, estimates suggest that the number of identifiable criminal organizations in Mexico grew from ~98 in 2010 to ~198 groups by 2019, although such counts vary by methods and definitions.^7^

 The immediate violence following El Mencho’s death should therefore be understood primarily as coordinated retaliation, not as direct evidence of organizational fragmentation. Read alongside broader evidence on leadership decapitation, however, the episode illustrates a recurring problem: even tactically successful operations can generate acute civilian harm while doing little to alter the conditions that allow criminal organizations to reconstitute, fragment, or escalate competition over time. The recent split within the Sinaloa Cartel following the capture of Ismael “El Mayo” Zambada similarly illustrates this dynamic, with rival *Mayiza* and *Chapitos* factions now waging war across Sinaloa.^8^

 None of this implies that cartel leaders should enjoy impunity. The gravity of their crimes, including mass murder, torture, enforced disappearances, human trafficking, and the large-scale trafficking of unregulated drugs, demands investigation and prosecution. However, the policy debate must distinguish clearly between seeking retributive justice and violence reduction. Conflating the two and treating kingpin removal as *both* punishment and strategy has produced neither lasting justice nor lasting peace.^6^ Criminal leaders and the broader command structures that enable them must be held accountable through strengthened judicial institutions, with force, where necessary, used to support investigation and prosecution rather than substitute for them.^9^

## The Political Economy of Cartel Power

 The failures of the kingpin strategy reflect a mismatch between the enforcement target and the structure of cartel power. Cartel power is not sustained by drug trafficking alone. It is reproduced through a wider system of illicit and semi-licit revenues, coercive territorial control, corruption, weak institutions, and transborder flows of drugs, weapons, money, and political incentives.^4,10^

 Mexico’s major criminal organizations, including CJNG, have diversified far beyond drug trafficking into extortion, fuel theft (*huachicol*), illegal mining and logging, human trafficking, kidnapping, and other forms of criminal extraction.^11^ For many communities, cartel power is experienced less through trafficking violence than through everyday criminal governance, such as extortion fees, protection rackets, forced recruitment, threats to small businesses, and the capture of local authorities.

 This diversification changes the meaning of violence-reduction policies. If criminal organizations derive power from multiple revenue streams and from their capacity to govern through fear, then removing a leader or reducing one illicit market cannot by itself dismantle the conditions that sustain violence. A realistic violence-reduction strategy must therefore target the organizational ecosystem that converts revenue into governance, including money laundering, sectoral infiltration, corruption, impunity, and community intimidation. Recent FinCEN guidance on bulk cash smuggling and repatriation by Mexico-based transnational criminal organizations underscores the importance of following financial flows across both the United States (US) and Mexican financial systems.^12^

 Equally critical is the role of corruption. Collusion between criminal organizations and state authorities at municipal, state, and federal levels is a structural enabler of cartel power and a driver of inter- and intra-organizational conflict.^13^ This problem intensified in the early 2000s during Mexico’s democratic transition, which disrupted the centralized, state-mediated protection arrangements of the *Partido Revolucionario Institucional* (Institutional Revolutionary Party).^14^ Mexico’s 2019 transformation of the Federal Police into the militarized National Guard has not resolved institutional weakness and has raised concerns about human rights abuses and the normalization of military roles in public security.^15^

 Uneven state capacity reinforces this political economy. State institutions may be able to mobilize quickly around high-profile enforcement actions while remaining weak, captured, or under-resourced in the domains that matter most for ordinary victims (eg, search, investigation, prosecution, witness protection, forensic identification, and prevention of recruitment). Mexican authorities were able to confirm and return El Mencho’s body to his family,^16^ yet families of disappeared persons continue to confront delayed searches, weak investigations, and overwhelmed forensic systems in a country where at least 115 571 people remained officially registered as disappeared or missing as of mid-2024.^17^ Recent research on disappearances in Mexico City highlights the relationship between disappearances, drug dealing, and organized crime, while modeling work on cartel recruitment suggests that violence reduction requires strategies that reduce recruitment rather than relying primarily on incapacitation.^18,19^ A public security strategy centred on spectacular arrests and killings therefore risks privileging visible state action over the slower work of search, justice, prevention, and institutional repair.

## Why Regulation Is Necessary but Insufficient

 Drug regulation can advance public health and reduce selected criminal rents by bringing particular drug markets under public oversight, reducing harms associated with prohibition, and supporting access to treatment and harm reduction. Regulation is therefore a necessary, though insufficient, component of a broader violence-reduction strategy. The public health case for this shift is well-established. The 2016 Lancet Commission on Drug Policy and Health concluded that enforcement-based approaches have worsened public health outcomes and recommended a gradual transition toward regulated drug markets.^20^

 Evidence from Canada and the US suggests that regulated markets can displace some illicit revenues under specific institutional conditions. Statistics Canada estimates from 2023 suggest that 73% of household expenditures on non-medical cannabis came from legal sources, while 79% of cannabis consumers reported always or mostly purchasing cannabis from legal sources.^21^ A study of US medical cannabis laws has also estimated a ~13% reduction in drug trafficking violence in border areas.^22^ These findings matter because they show that legal markets can alter the relationship between consumer demand and criminal revenue.

 Regulatory design should nevertheless be substance-specific. Cannabis can be governed through public-health-oriented controls on product quality, marketing, price, and access, whereas opioid markets require more tightly controlled, health-centred models embedded within treatment, overdose prevention, and harm reduction systems. The relevance of such approaches to Mexico, however, is limited by institutional context and cartel diversification. Cannabis is no longer the principal revenue source for major Mexican criminal organizations,^23^ and legal markets require credible state capacity, protection of legal market participants, anti-corruption safeguards, and mechanisms to prevent criminal infiltration. In Mexico, the Supreme Court ruled the absolute prohibition of cannabis use unconstitutional, yet Congress has failed to enact the regulatory legislation, echoing the implementation failures that undermined Mexico’s landmark 2009 drug decriminalization reforms.^24,25^

 The rise of synthetic drug markets underscores the limits of prohibition and the need for public-health-oriented regulation. Prohibition and enforcement pressure can favor compact, high-potency products that are easier to transport and conceal, while unregulated markets expose people to unpredictable potency and adulteration.^20^ Fentanyl and methamphetamine production and trafficking networks operating in Mexico are tightly linked to the ongoing overdose crisis in the US, which claims tens of thousands of lives annually.^26^ These synthetic drugs now represent a significant and growing share of cartel revenue,^11^ and require responses calibrated to their risks, dependence potential, and market dynamics rather than a single regulatory model. In highly adaptive synthetic drug markets, enforcement strategies that destabilize trafficking hierarchies can intensify market volatility when they are not accompanied by demand reduction, treatment access, and harm reduction. The result may be greater unpredictability in drug supply and increased overdose risk in consuming countries.^27^ These interconnected harms require strengthened regional coordination to align public health, regulatory, and criminal justice responses across borders.

 Regulation, therefore, can reduce drug-market-derived criminal rents, but it cannot dismantle the diversified revenue streams, coercive local control, corruption, and institutional weakness through which cartel power is sustained. Its contribution is greatest when paired with treatment and harm reduction, financial investigation, anti-extortion capacity, sectoral oversight, victim protection, and institutional reform.

## Reclaiming State Capacity Without Reproducing Militarization

 Reclaiming territorial control and dismantling parallel criminal governance require more than militarized decapitation. The state has an obligation to protect civilians, restore lawful authority, and dismantle coercive local rule. A more credible approach would sequence state action around durable civilian protection rather than spectacular leadership removal.

 In practice, this means strengthening the protective, investigative, judicial, and preventive functions of the state.^28^ Protective capacity includes preventing extortion, safeguarding small businesses and local officials, and protecting victims and witnesses from retaliation.^29^ Extortion and protection rackets have become a major source of insecurity for businesses and communities in Mexico, while witness protection is widely recognized as essential in organized-crime proceedings. Investigative and prosecutorial capacity includes intelligence-led investigations, prosecutorial independence, forensic identification, anti-corruption enforcement, and credible prosecution of violent actors and the networks that enable them. Judicial capacity requires courts capable of converting investigations into lawful accountability rather than allowing impunity to prevail. Preventive capacity includes reducing recruitment into criminal organizations through social and economic measures, especially in territories where criminal groups function as employers, governors, and coercive authorities.^18^

 This distinction is central to Mexico’s sovereignty problem.^13^ In parts of Mexico where criminal organizations exercise de facto authority, neither regulatory reform nor symbolic demonstrations of force can restore safety on their own. Reclaiming sovereignty requires rebuilding everyday capacities that make lawful authority credible—from preventing extortion and investigating disappearances to limiting corruption and preventing forced recruitment.^30-32^

 Mexican scholars and practitioners have long advocated alternative models centred on community-oriented policing, police accountability, and local institutional strengthening drawing on comparative experiences (eg, Colombia’s professionalized national police).^33^ These approaches are less dramatic than military operations, but they address the governance deficits that allow cartels to co-opt state institutions and operate with near-impunity. Force may sometimes be necessary to protect communities; however, its legitimate role should be measured by whether it supports civilian protection, prosecution, and durable institutional control, rather than by the visibility of arrests or killings.

## Cooperation Without Subordination: Beyond the US–Mexico Blame Cycle

 The persistence of the kingpin strategy also reflects bilateral political incentives. High-profile captures and killings serve as visible signals of cooperation with the US, where security assistance, extradition priorities, and diplomatic pressure have often rewarded spectacular enforcement actions. These incentives can remain powerful even when their domestic consequences include retaliation, fragmentation, or civilian harm. Bilateral cooperation, however, should not be judged by highly visible enforcement alone, but by whether it reduces the harms experienced by communities on both sides of the border.

 Mexican institutional reform cannot, on its own, offset the availability of firearms trafficked from the US. Bureau of Alcohol, Tobacco, Firearms and Explosives trace data require cautious interpretation because firearms recovered in Mexico and submitted for tracing are not a random sample of all firearms used in crime. Nevertheless, these data indicate that the US is a major source country for firearms recovered in Mexico and submitted for tracing.^34^ Some policy sources have estimated that 70%–80% of cartel firearms, particularly high-powered assault rifles, originate in the US, with annual estimates ranging from 200 000 to 500 000 weapons illegally trafficked southward.^35,36^ Without stronger US measures to prevent diversion from lawful commerce and improved bilateral efforts to disrupt firearms trafficking, cartels will retain the firepower to challenge the state, intimidate communities, and outmatch local authorities.

 The same principle applies to drug demand and overdose risk. Synthetic drug markets link production, trafficking, consumption, and mortality across borders. Enforcement actions in Mexico cannot be evaluated separately from demand, treatment access, harm reduction, and overdose prevention in the US. A bilateral strategy focused on arrests, extraditions, and seizures is incomplete when it neglects firearms flows, drug demand, public health, and institutional reform.

 Because this is a cross-border crisis, the politics of blame is analytically and politically inadequate. Mexican discourse often centres on US weapons and drug consumption, while US discourse emphasizes Mexican production, trafficking, and corruption. Both narratives contain truth; neither captures the full system. Cooperation without subordination requires reciprocal accountability: Mexico must strengthen civilian protection, justice institutions, anti-corruption enforcement, and recruitment prevention; the US must address firearms diversion, drug demand, treatment access, harm reduction, and overdose prevention. Success should be measured not by arrests or seizures alone, but by reductions in violence, extortion, disappearances, recruitment, firearms trafficking, overdose mortality, and impunity.

## A Path Forward

 The violence following El Mencho’s death should caution against both the failed simplicity of the kingpin strategy and the temptation to replace it with another single-solution paradigm. Leadership decapitation may produce tactical successes, but lasting violence reduction requires action on the structures that reproduce criminal governance.

 The path forward requires a holistic political economy approach to cartel violence reduction: carefully sequenced regulation where feasible; substance use treatment and harm reduction; disruption of diversified criminal economies; strengthened civilian policing, prosecutorial capacity, and judicial institutions; protection from extortion, disappearance, and forced recruitment; anti-corruption enforcement; and reciprocal US–Mexico cooperation and accountability. The choice ahead is between continuing a paradigm that produces cycles of retaliation and cartel reorganization, and building the institutional, public health, and regional capacity that genuine violence reduction demands. The hundreds of thousands of lives lost in Mexico’s drug war demand nothing less.

## Acknowledgment

 Mohammad Karamouzian is supported by the Peterson Foundation and Aune Foundation.

## Disclosure of artificial intelligence (AI) use

 During the preparation of this work, the authors used Grammarly to check for spelling, grammar, punctuation errors, and to refine language. The authors also used ChatGPT V.5.5 to assist with the development of the figure. After using these tools, the authors reviewed, edited, and verified the content as needed and take full responsibility for the content of the publication.

## Ethical issues

 Not applicable.

## Conflicts of interest

 Authors declare that they have no conflicts of interest.
